# Hepatoprotective and in vivo antioxidant effects of granulometric classes and decoction of *Ficus dicranostyla* Mildbread leaves powders against carbon tetrachloride‐induced hepatotoxicity in Wistar rats

**DOI:** 10.1002/fsn3.3582

**Published:** 2023-08-23

**Authors:** Yves Tabi Omgba, Marthe Valentine Tsague, Romeo Joël Temdie Guemmogne, Achick Estella Tembe, Rose Ngono Mballa, Ntungwen Charles Fokunang, Bonaventure Ngadjui Tchaleu, Théophile Dimo, Judith Ndongo Embola, Jacqueline Ze Minkande

**Affiliations:** ^1^ Department of Pharmacotoxicology and Pharmacokinetics, Faculty of Medicine and Biomedical Sciences University of Yaoundé I Yaounde Cameroon; ^2^ Department of Biomedical Sciences, Faculty of Sciences University of Ngaoundere Ngaoundere Cameroon; ^3^ Department of Biological Sciences, Faculty of Sciences University of Ngaoundere Ngaoundere Cameroon; ^4^ Department of Pharmacology and Traditional Medicine, Faculty of Medicine and Biomedical Sciences University of Yaoundé I Yaounde Cameroon; ^5^ Department of Chemistry, Faculty of Sciences University of Yaounde I Yaounde Cameroon; ^6^ Department of Physiology, Faculty of Sciences University of Yaounde I Yaounde Cameroon; ^7^ Department of Physiological Sciences/Biochemistry, Faculty of Medicine and Biomedical Sciences University of Yaoundé I Yaounde Cameroon; ^8^ Department of Surgery and Specialties, Faculty of Medicine and Biomedical Sciences University of Yaoundé I Yaounde Cameroon

**Keywords:** antioxidant, *Ficus dicranostyla*, hepatoprotective, powder

## Abstract

*Ficus dicranostyla* is a plant from the Moraceae family commonly used in African countries for its nutritional value and its believed medicinal properties. Its antioxidant in vitro capacity and its richness in phenolic compounds have been previously demonstrated. This work aimed at evaluating the hepatoprotective and in vivo antioxidant activities of different granulometric fractions of the *F. dicranostyla* leaves against carbon tetrachloride‐induced hepatotoxicity in rats. Powdery fractions (<125, 250–125, and ≥250 μm), and the unsieved powder, obtained from the *F. dicranostyla* leaves were water‐dissolved and given orally to rats at the same dose (250 mg/kg body weight) before administering carbon tetrachloride intraperitoneally (1 mL/Kg bw). The lipid status parameters (total cholesterol, triglycerides, HDL‐cholesterol, and LDL‐cholesterol), hepatic toxicity through aspartate aminotransferase (ASAT) and alanine aminotransferase (ALAT) in blood plasma, and antioxidant status by measuring the malondialdehyde (MDA), superoxide dismutase (SOD), and catalase (CAT) in liver homogenate were performed. The activities of all parameters registered a significant (*p* < .05) alteration in CCl_4_‐treated rats, which were significantly recovered toward an almost normal level in coadministered with *Ficus dicranostyla* leaf powder samples in a particle size‐dependent manner. Results suggest that the smaller particle size of the powder fraction, as well as the decoction powder of *Ficus dicranostyla,* may be used as hepatoprotective and antioxidant agents.

## Graphical abstract

The aim of this article is to evaluate the preventive, hepatoprotective effects of granulometric classes, unsieved powder and decoction fraction of *Ficus dicranostyla* leaf powders against carbon tetrachloride‐induced hepatotoxicity in rats.

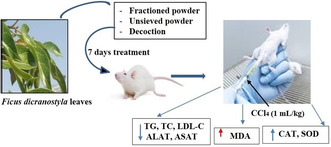

## 1 INTRODUCTION

Chronic liver diseases (CLD) constitute one of the most prevalent diseases in the world (Shantan Cheemerla & Maya Balakrishnan, 2021). Although improved, prevention strategies and treatment (in the case of hepatitis) have led to improving CLD trends, available pharmacotherapeutic options for liver disease produce limited results with accompanying side effects. The use of herbs in complementary and alternative medicine has sparked research interest in new plausible hepatoprotective agents capable of ameliorating or reversing liver damage with few side effects (Ugwu & Suru, 2021). Over the years, research gained an interest in effective natural products/drugs, and whose natural plant is one of the sources (Chawda et al., 2017). *Ficus dicranostyla* is a plant species from the Moraceae family found in savannas and galleries in Sudanese and Guinean forests (Diop, 2006) and is among such natural resources. It is widely distributed in various African countries including; Cameroon, Ethiopia, Uganda, and Zambia (Diop, 2013; Loutfy et al., 2005). All parts of the plant are used for their nutritional value and supposed medicinal properties similar to that of other leaves of *Ficus* species (Zaitun et al., 2012). *Ficus dicranostyla* leaves and barks are part of delicious traditional dishes in the Far North region of Cameroon (Malzy, 1954). The biological properties and medicinal functions of *F. dicranostyla* leaf extracts were mainly established by in vitro assays based on their antioxidant capacity and their richness in phenolic compounds (Yao et al., 2014). This study shows the antioxidant activity of this plant attributed to its total polyphenol content. Extraction is the initial step to use the active compounds of the plant matrix. Generally, the use of plants as functional foods is based on traditional practices (powders, aqueous, or dry extracts) and modern extraction methods (developed more recently) designed for the extraction of bioactive compounds from plants (Deli, Ndjantou, et al., 2019). Conventional methods (percolation, maceration, decoction, soxhlet extraction, and hydrodistillation) are mainly based on the use of mild/high temperatures (50–90°C) that can cause thermal degradation. Long duration, large volumes of organic solvents and/or water, high costs of these extraction procedures, and sometimes low extraction yield are the drawbacks of conventional methods (Lezoul et al., 2020; Noore et al., 2021; Palmade‐Le Dantec & Picot, 2010). Today, extraction methods are continuously improved due to these drawbacks. Less expensive and more environmentally friendly extraction processes are preferred to isolate bioactive compounds from plants, fruits, or vegetables.

In this context, Baudelaire (2013) implemented a controlled differential sieving process (CDSp) to concentrate active compounds from plant matrices.

Indeed, the treatment of the plant matrix by this technology permits to improve the activity and concentrate bioactive compounds including phenolic compounds, vitamins, minerals, and essential oils (Baudelaire, 2013; Becker et al., 2016, 2017; Deli, Baudelaire, et al., 2020; Deli, Ndjantou, et al., 2019; Deli, Nguimbou, et al., 2020; Deli, Petit, et al., 2019; Mbassi et al., 2021; Noumi et al., 2021; Soualeh, Stiévenard, Baudelaire, Bouayed, & Soulimani, 2018; Soualeh, Stiévenard, Baudelaire, Soulimani, & Bouayed, 2018; Zaiter et al., 2016). The advantage of using grinding followed by CDSp is the possibility to easily optimize the extraction conditions of bioactive compounds from plants.

Therefore, the present study was conducted to evaluate the antioxidant and hepatoprotective activities of granulometric classes and decoction of *F. dicranostyla* against carbon tetrachloride‐induced hepatotoxicity in rats.

## 2 MATERIALS AND METHODS

### 2.1 Plant material

Fresh tender leaves of *Ficus dicranostyla* Mildbread (Collector: R. Letouzey number 6951; Herbarium number 8618 SRF/Cam) were collected in July 2021 from the Mokolo local area of Maroua, Cameroon, capital of the Far‐ Nord Region (Cameroon). Once at the laboratory, the leaves were cleaned and washed in potable water to remove dust, dirt, and any other type of sticky material and used for further processing. Then, leaves were kept on sieves to drain out excess water and were air‐dried in a ventilated oven at 40°C for 24 h to pulverize into a fine powder suitable for sieved fractionation.

### 2.2 Grinding of dried leaves

Obtained dried leaves of *Ficus dicranostyla* were finely ground in an electric BIOBASE Disintegrator grinder (Model MPD‐102, N°: 61 South Gongye Road Jinan City, China; Serial N°: 20020020) supplied with a sieve drilled with 1 mm trapezoidal holes. The grinding operation was operated at 1400 rpm/min for 1 min in ambient laboratory air. Produced *Ficus dicranostyla* leaf powder was divided into three lots: the first lot of unsieved powder, was kept to serve as a control. The second lot of powder corresponds to sieved powders and the third lot was used to prepare solvent extract using the conventional decoction method.

### 2.3 Sieve fractionation of powder

Produced fine powder from *Ficus dicranostyla* leaves was separated into three powder fractions based on the particle size using two sieves of different mesh sizes. Practically, two selected sieves of 125 and 250 μm apertures were installed on MINOR sieve shaker operating by vertical vibration à 0.5 mm vibration amplitude. For each batch, 50 g of mother leaf powder was poured on the top sieve and a controlled differential sieving process (CDSp) was performed in the permanent mode for 15 min. After that, the powder mass retained on each sieved was collected and weighed. This permitted the production of three powder fractions according to their particle sizes: less than 125 μm (called powder fraction of <125 μm), between 125 and 250 μm (called powder fraction of 125–250 μm), and greater than 250 μm (referred as powder fraction ≥250 μm). The mass or quantity of powder retained on each sieve was collected and then stored in polyethylene bottles and in darkness until they were used.

### 2.4 Preparation of the aqueous extract using the decoction method

The decoction was performed by mixing a mass (80 g) of *Ficus dicranostyla* leaf powder in 800 mL of distilled water contained in a 1L beaker (1/10 w/v). The mixture was put in a boiling water bath for 30 min and subjected to manual agitation using a spatula every 5 min. Then, the mixture was left to stand for 5 min and then filtered through Whatman N°1 filter paper. The obtained decoction was kept in a freezer at −18°C for 24 h and finally was freeze‐dried (Christ®, alpha 1–2 LD) at −60°C for 48 h. Freeze‐dried collected extract, called decoction fraction was conditioned in polyethylene bottles until further analysis.

### 2.5 Animals

In this experiment, male Albinos Wistar rats (*Rattus norvegicus*) with body weights between 250 and 300 g and 3 months old were used. The inbreed colonies of rats were raised at the animal house of the Laboratory of Biophysics, Food Biochemistry and Nutrition (LABBAN) of Ngaoundere University, Cameroon under the following: rats were maintained under room temperature at 25 ± 4°C, 12 h light, and 12 h dark and free access to food and water allowed *ad libitum* (Ngatchic et al., 2013).

### 2.6 Treatment design

The experiment was conducted on 35 male Wistar rats. After acclimation, the rats were randomly divided into the following groups with five rats in each group and per cage:

Group 1: Negative control or hepatotoxic control rats received CCl_4_ at 1 mL/kg bw (body weight) only, intraperitoneally.
Group 2: Normal healthy control rats received distilled water at 1 mL/kg bw only, intraperitoneally.
Group 3–7: Treatment rat groups received CCl_4_ at 1 mL/kg/bw, intraperitoneally and 250 mg/kg bw per os of different powder fractions (<125, 125–250, and ≥250 μm), unsieved powder, and decoction fraction, respectively.

All the animals were treated as shown above for 7 days. Before foods were given to rats, each powder sample was previously dissolved in distilled water by stirring using a magnetic stirrer at 3500 rpm for 12 h. Distilled water (used to treat negative control and normal control rat groups) and macerated plant powder samples (used for experimental rat groups) were administered every day for 7 days. On the seventh day, 1 h after the last dose of powder fraction solution or distilled water, rats were treated intraperitoneally with CCl_4_ previously dissolved in olive oil (1:1, v/v). Rats were fed on a basal diet or normal diet and water, as a proposed protocol by Ngatchic et al. (2013).

#### 2.6.1 Preparation of liver homogenate and blood plasma

Twenty‐four hours after the administration of CCl_4_, all the rats were anesthetized using a glass jar containing cotton wool that was soaked in diethyl ether. Immediately, 1–2 mL of blood samples were collected in heparin tubes after cardiac puncture and were subjected to centrifugation (3500 rpm for 15 min) at 4°C to obtain the plasma which was kept frozen at −4°C until they were used for determination of lipid status markers and transaminases. After the collection of blood samples, the rats in different groups were dissected and their livers were excised immediately from three rats in each group; and washed immediately with ice‐cold saline to remove as much blood as possible. The liver homogenate was prepared from 1 g of liver tissues which were homogenized in a China mortar with phosphate buffer (0.1 M pH 7.4 having 0.15 M KCl). The liver homogenate was subjected to centrifugation (3500 rpm for 15 min) and the supernatant was collected and kept frozen at −4°C until used to determination of oxidative stress markers.

#### 2.6.2 Biochemical analysis

##### Measurement of lipid profile in blood plasma

Using commercialized available kits and based on the established spectrophotometric methods, according to the manuals supplied, the parameters of the lipid status were determined by measuring the levels of triglyceride (TG) by enzymatic method CPO‐PAP, total cholesterol (CT) using enzymatic colorimetric test for cholesterol with liquid clearing factor (Human, Max‐Planck‐Ring 21. 65,205 Wiesbaden. Germany), high‐density lipoprotein (HDL‐c) cholesterol using a kit (CHRONOLAS SYSTEMS, S.L., Travessia Prat de la Riba 34 B, 08849 Sant Climent de Llobregat, Barcelona, Spain) according to the method of Naito (1984) and low‐density lipoprotein (LDL‐c) cholesterol was deduced from the following formula:

$$\mathrm{LDL}‐cholesterol=\text{total cholesterol}-\mathrm{HDL}‐cholesterol\;–\;\left(\mathrm{TG}/5\right).$$

##### Measurement of liver function markers in blood plasma

Liver functions were evaluated by measurement of the activity of the enzymes alanine aminotransferase (ALAT) and aspartate aminotransferase (ASAT), which mark hepatocellular lesions. ASAT and ALAT activities were performed using commercially available kits based on the established spectrophotometric methods, according to the supplied manuals (Bergmeyer et al., 1986; Henry et al., 1960, respectively).

##### Evaluation of CCl_4_‐mediated oxidative stress

###### Measurement of SOD and CAT activities

To further assess oxidative stress, the activities of the antioxidant enzymes including catalase (CAT) and superoxide dismutase (SOD) were determined in the liver homogenates of the control and experimental rat groups according to the methods of Sinha (1972) and Beauchamp and Fridovich (1971), respectively.

For CAT determination, 1 mL of phosphate buffer (0.1 M, pH 7.4) and 0.4 mL of 0.2 M H_2_O_2_ were added to 100 μL of liver homogenate contained in the tube. The reaction was stopped at 30, 60, and 90 s by adding 2 mL of dichromate/acetic acid mixture (5:95, v/v). Absorbance was measured at 620 nm, and CAT activity was expressed in units per milligram of protein using a molar extinction coefficient of CAT (*ε* = 0.036 mmol‐1.cm^−1^).

For measurement of SOD activity, 2.5 mL of 0.1 M carbonate buffer solution at pH 10.2 was added to 0.2 mL of liver homogenate. Then, 0.3 mL of adrenalin solution prepared at 5 μg/mL in water was added to the mixture to initiate the reaction of conversion of adrenaline to adrenochrome and the whole was vortexed. Absorbance was measured every 30 s until 150 s in order to follow an increase of absorbance at 480 nm. A volume of 0.3 mL of distilled water was used in a reference tube. Calculated SOD activity was expressed as units per milligram of protein.

###### Determination of tissue lipid peroxidation

The extent of lipid peroxidation (LPO) was estimated in the liver homogenate of all rats as the concentration of thiobarbituric acid reactive product (Malondialdehyde‐MDA) by the spectrophotometric method (Yagi, 1976). A volume of 100 μL of homogenate liver, 400 μL of TBA reagent, and 80 μL of HCl were successively introduced into a test tube. The mixture was vortexed and incubated in a boiling water bath for 15 min. After cooling in a cold water bath for 30 min, the mixture was centrifuged at 603 *g* for 15 min. The absorbance of the collected supernatant was read at 530 nm using a ultraviolet–visible spectrophotometer. Results were expressed as malondialdehyde (MDA) content in μmol/mg protein using a molar extinction coefficient of MDA (*ε* = 1.56, 105 mol/L^−1^.cm^−1^).

### 2.7 Statistical analysis

The obtained data were recorded in an Excel file, and analysis was carried out in triplicate. The experimental results were reported as mean ± standard error mean deviation from three repeated determinations. Analysis of comparison between groups was performed statistically using one‐way analysis of variance (ANOVA), followed by Duncan's multiple range test performed by Statgraphics to determine significant differences among the samples or intergroup variation, and was considered as significant difference at *p* <.05.

## 3 RESULTS

### 3.1 Effect of particle size fractions, unsieved powder, and decoction fraction on lipid status

Table 1 presents the effect of different granulometric classes, unsieved, and decoction powders from *F. dicranostyla* leaves on the blood plasma lipid profile in CCl_4_‐induced injury in rats. A significant difference (*p* < .05) between the different treatment groups in terms of their liver CT, TG, HDL‐c, and LDL‐c levels was noted. Rats subjected to the CCl_4_ challenge alone (negative control rats) developed significant liver injury as evident from a significant increase in plasmatic lipid accumulation markers, namely, CT, TG, and LDL‐c levels to 180.15, 209.10, and 129.75 mg/dL, respectively, whereas that of normal control rats were only 72.65, 76.85, and 29.64 mg/dL, respectively. On the other hand, the HDL‐c level observed in negative control rats (8.58 mg/dL) was significantly decreased compared to those of normal control rats (27.64 mg/dL).

**TABLE 1:** Effect of particle size fractions, unsieved powder, and decoction from *F. dicranostyla* leaves on the lipid status.

Rat groups	CT	TG	HDL‐c	LDL‐c
Normal control	72.65 ±5.15^a^	76.85 ± 0.55^a^	27.64 ± 1.89^e^	29.64 ± 3.32^a^
Negative control	180.15 ± 5.15^e^	209.10 ± 6.90^d^	8.58 ± 0.81^a^	129.75 ± 6.76^e^
<125 μm	78.25 ± 7.25^ab^	85.75 ± 10.15^a^	23.08 ± 1.52^d^	38.02 ± 7.47^b^
125–250 μm	95.80 ± 4.36^c^	106.73 ± 5.70^b^	20.47 ± 1.86^c^	53.98 ± 2.64^c^
≥250 μm	138.80 ± 1.90^d^	139.95 ± 9.15^c^	14.69 ± 0.64^b^	96.12 ± 1.78^d^
Unsieved powder	96.03 ± 5.54^c^	105.10 ± 4.80^b^	17.04 ± 0.73^b^	60.99 ± 1.16^c^
Decoction	81.90 ± 2.90^b^	83.40 ± 4.90^a^	23.23 ± 1.47^d^	41.99 ± 2.41^b^

*Note*: Values in the same column with different superscripted letters differed significantly (*p* < .05) according to Duncan's multiple range test (*n* = 3).

Abbreviations: CT, total cholesterol; HDL‐c (mg/dL), high‐density lipo‐cholesterol; LDL‐c (mg/dL), low‐density lipo‐cholesterol; TG (mg/dL), triglycerides.

Treatment with different granulometric classes, unsieved, and decoction powders of *F. dicranostyla* significantly (*p* <.05) inhibited the accumulation of CT, TG, and LDL‐c levels in the plasma of rats, while HDL‐c levels inversely increased. It was also noted as a significant (*p* < .05) effect of powder particle sizes on these lipid status markers. While powder fraction of <125 μm and decoction had the higher inhibitory activity (lower blood plasma CT, TG, and LDL‐c levels, and inversely higher HDL‐c level), the powder fraction of 125–250 μm and unsieved powder followed, and the powder fraction of ≥250 μm ended. The administration of *F. dicranostyla* powders to rats treated with CCl_4_ protected them against lipid accumulation and in some treatments such as powder fraction of <125 μm and decoction powder, the protection was 100% associated with improvement of TG, CT, and LDL‐c levels. More important to mention is the HDL‐c levels which significantly increased in the treatments with powder fraction of <125 μm and decoction powder. A decrease in TG, CT, and LDL‐c levels was equally observed with the administration of *F. dicranostyla* powder fraction of 125–250 μm and unsieved powder followed and powder fraction of ≥250 μm, but to a lesser extent, that of <125 μm and decoction fractions.

### 3.2 Effect of particle size fractions, unsieved powder, and decoction fraction on liver function parameters

**FIGURE 1:**
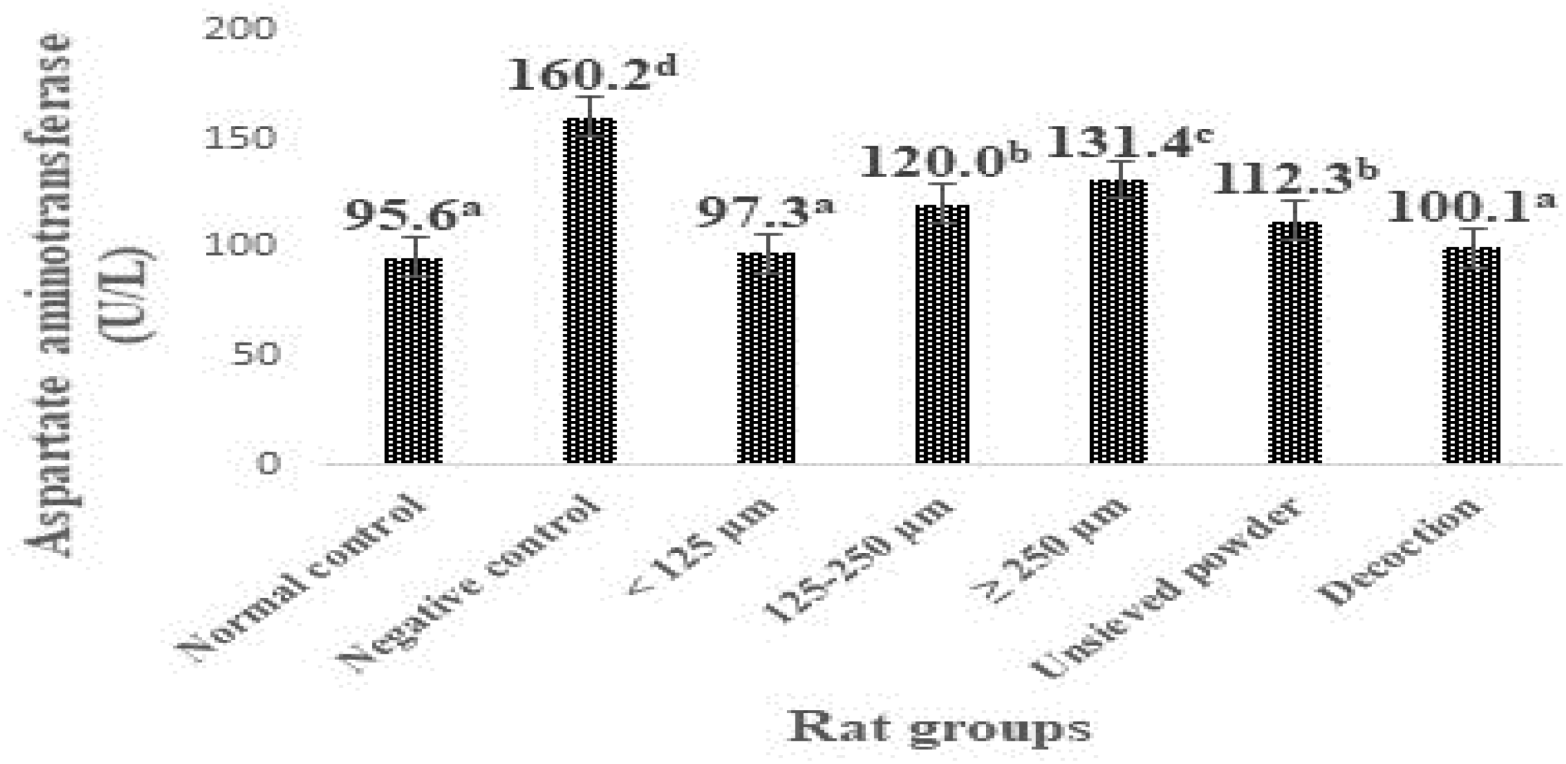
Effect of particle size fractions, unsieved powder, and decoction from *F. dicranostyla* leaves on the activity of ASAT (aspartate aminotransferase) in normal and CCl_4_‐treated rats. <125 μm: Powdery fractions with a diameter inferior at 125 μm; 125–250 μm: Powdery fractions with a diameter between 125 and 250 μm ≥250 μm: Powdery fractions with a diameter superior at 250 μm; U/L: units per liter. Bars with different superscripted letters differed significantly (*p* < .05) according to Duncan's multiple range test (*n* = 3).

**FIGURE 2:**
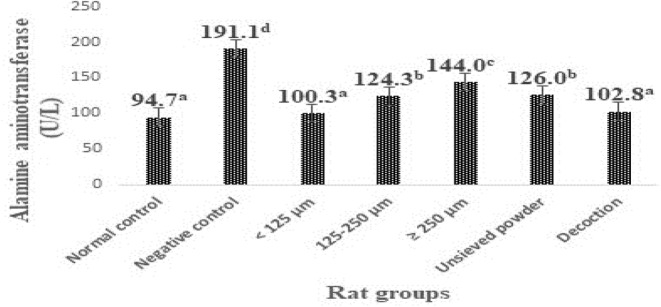
Effect of particle size fractions, unsieved powder, and decoction from *F. dicranostyla* leaves on the activity of ALAT (alanine aminotransferase) in normal and CCl_4_‐treated rats. <125 μm: Powdery fractions with a diameter inferior at 125 μm; 125–250 μm: Powdery fractions with a diameter between 125 and 250 μm ≥250 μm: Powdery fractions with a diameter superior at 250 μm; U/L: units per liter. Bars with different superscripted letters differed significantly (*p* < .05) according to Duncan's multiple range test (*n* = 3).

The levels of transaminases in the liver of animals in the different groups are shown in Figures 1 and 2. Rats subjected to the CCl_4_ challenge alone (negative control rats) developed significant (*p* < .05) liver toxicity as evident from a significant elevation in ALAT and ASAT levels (160.2 and 191.1 U/L, respectively) compared with normal control rats (95.6 and 94.7 U/L, respectively).

It was also noted from Figures 1 and 2 that the treatments with different granulometric classes, unsieved powder, and decoction powder from *F. dicranostyla* leaves at a dose of 250 mg/kg of body weight caused a significant decrease (*p* < .05) in the blood plasma ASAT and ALAT levels of the all treatment rat groups as compared to negative control rats. In particular, the powder fraction of <125 μm and decoction powder almost completely restored the ALAT and ASAT activities (100.3–97.7 U/L and 102.8–100.1 U/L, respectively) to the control levels (94.7 and 95.6 U/L, respectively). Indeed, no significant difference was observed between ALAT and ASAT activities of rat groups that received powder fraction of <125 μm and those of rat groups that received decoction powder compared to those normal control rats. On the other hand, the intermediate powder fraction of 125–250 μm, unsieved powder, and larger powder fraction of ≥250 μm also inhibited an increase in ALAT and ASAT activities or lipid peroxidation, but to a lesser extent than that of <125 μm and decoction fractions.

### 3.3 Effect of particle size fractions, unsieved powder, and decoction fraction on oxidative stress parameters

#### 3.3.1 Lipid peroxidation

From the results presented in Table 2, intraperitoneally CCl_4_‐induced in rats without treatment (negative control rats) caused a significant increase in the MDA concentration (56.13 μmol/mg of protein) compared to the normal control rats (15.83 μmol/mg of protein). Treatment with different granulometric classes, unsieved, and decoction powders from *F. dicranostyla* leaves at a dose of 250 mg/kg with the CCl_4_ induction caused a significant decrease (*p* < .05) in the MDA concentration of all treatment rat groups as compared to negative control rats. The results show that liver MDA concentration was significantly reduced in the rats treated with powder fraction of <125 μm (14.15 μmol/mg), followed by decoction and 125–250 μm fraction (20.52 and 21.60 μmol/mg, respectively), compared to the rats which received the solution of unsieved powder and powder fraction of ≥250 μm reduced lesser. Interestingly, no significant difference was observed between the normal control rats and the rat group that received a solution of <125 μm fraction.

**TABLE 2:** Effect of particle size fractions, unsieved powder, and decoction from *F. dicranostyla* leaves on liver SOD, CAT, and MDA in normal and CCl_4_‐treated rats.

Rat groups	SOD activity	CAT activity	MDA concentration
Normal control (NC)	364.71 ± 13.44^e^	343.29 ± 3.27^e^	15.83 ± 1.61^ab^
Negative control	151.59 ± 1.86^a^	117.92 ± 14.10^a^	56.13 ± 5.36^e^
<125 μm	304.99 ± 3.78^d^	316.83 ± 5.06^de^	14.15 ± 0.30^a^
125–250 μm	259.15 ± 4.66^c^	267.00 ± 15.62^c^	21.60 ± 0.94^c^
≥250 μm	212.10 ± 2.08^b^	208.33 ± 7.09^b^	33.41 ± 4.13^d^
Unsieved powder	298.90 ± 2.57^d^	226.82 ± 4.34^b^	23.68 ± 2.91^c^
Decoction	301.69 ± 11.69^d^	294.93 ± 11.82^cd^	20.52 ± 2.03^bc^

*Note*: Values in the same column with different superscripted letters differed significantly (*p* < .05) according to Duncan's multiple range test (*n* = 3).

Abbreviations: CAT (UI/mg of protein): catalase; MDA (μmol/mg of protein): malondialdehyde; SOD (UI/mg of protein): superoxide dismutase.

#### 3.3.2 Superoxide dismutase and catalase activities

Table 2 shows the liver SOD and CAT activities. From the results, the injection of CCl_4_ without any treatment caused a significant (*p* < .05) decrease in CAT and SOD activities of the negative control rats (117.92 and 151.59 UI/mg of protein, respectively) when compared with the normal control rats (343.29 and 364.71 UI/mg of protein, respectively).

It can be observed that treatment with different granulometric classes, unsieved, and decoction from *F. dicranostyla* leaf powders at the dose of 250 mg/kg significantly (*p* < .05) CAT and SOD activities compared to negative control rats. Considering the granulometric classes or powder fractions, CAT and SOD activities were significantly (*p* < .05) affected by particle size. The highest liver CAT and SOD activities were observed in rat groups treated with finer powder fraction of <150 μm (316.83 and 304.99 UI/mg of protein, respectively), while the lowest CAT and SOD activities were observed for larger powder fraction of ≥250 μm (208.33 and 212.10 UI/mg of protein, respectively). Indeed, CAT and SOD activities of <125 μm fraction were similar or else near to those of normal control rats and those of rat groups that received decoction fraction (294.99 and 301.69 UI/mg of protein, respectively). The intermediate powder fraction of 125–250 μm and unsieved powder also inhibited lipid peroxidation but to a lesser extent than that of <125 μm and decoction fractions.

## 4 DISCUSSION

Studies have shown that *Ficus dicranostyla*, which is widely used for its nutritional value and supposed medicinal properties, possesses antioxidant properties in vitro, due to its richness in phenolic compounds. The aim of this work was to evaluate the in vivo hepatoprotective and antioxidant activities of different granulometric fractions of *F. dicranostyla* leaves against carbon tetrachloride‐induced hepatotoxicity in rats. Results showed that this plant significantly inhibited transaminase release and reduced oxidative stress induced by CCl_4_ administration in rats.

The liver is the main site of cholesterol metabolism. To a lesser extent, the adrenal cortex and gonads also contribute. Cholesterol is a general indicator of the level of atherogenic lipids in circulation, and cholesterol and polyunsaturated fatty acids are the main components of LDL‐c. Low levels of HDL‐c are also an important risk factor for cardiovascular disease (Helmy et al., 2017). The results showed a significant increase in total cholesterol (CT), triglycerides (TG), and LDL‐cholesterol levels in the blood plasma of CCl_4_‐treated rats, while at the same time, HDL‐cholesterol levels were significantly low. These observations could be attributed to CCl_4_‐induced hepatocyte damage. Treatment with unsieved *F. dicranostyla* powders, different particle size classes, and decoction significantly reduced plasma TC, TG, and LDL‐c levels, and increased HDL‐c levels. Fraction <125 μm and decoction demonstrated a higher protective effect against CCl_4_ hepatotoxicity. However, statistical analysis shows that the fraction smaller than 125 μm was slightly more effective than decoction on the lipid profile. In general, with regard to lipid status, our results demonstrated the hepatoprotective effects of *F. dicranostyla* leaf powders and suggest that they may protect against dyslipidemia and reduce the risk of cardiovascular disease (Itoro Usoh et al., 2012; Mckenney, 1999).

Since the liver is the major site of cholesterol metabolism with the intestine, adrenal cortex, and gonads making lesser contributions, the high blood plasma concentration of total cholesterol evident in CCl_4_‐treated groups might be attributed to the damage inflicted on the liver hepatocytes by this toxicant. Cholesterol is a general indicator of the level of atherogenic lipids in the circulation and cholesterol and polyunsaturated fatty acids are the main components of LDL‐c. A low level of HDL‐c is also an important risk factor for cardiovascular diseases (Helmy et al., 2017). A difference in analyzed blood plasma lipids according to particle size is probably a consequence of the unequal distribution of bioactive molecules during the sieved fractionation of powder. The high HDL‐c levels shown in our results in the *F. dicranostyla* leaf powders treated groups are desirable and the relationship between high HDL‐C levels and reduced cardiovascular risk has been reported (Itoro Usoh et al., 2012; Mckenney, 1999). In general, with regard to lipid status, these above results confirm the strong hepatoprotective effects of *F. dicranostyla* leaf powders and suggest that they could protect against lipid accumulation, their hepatoprotective effectiveness, and may also benefit in the context of the risk of liver diseases.

Our results show a significant increase in ALAT and ASAT levels in the negative control compared with the normal control. These results suggest that free radicals from CCl_4_ metabolism caused peroxidation of hepatocyte membranes, leading to their lysis. After treating the animals with decoction, unsieved powders, and fractions of *Ficus dicranostyla* powders, we recorded a significant drop in ALAT and ASAT activities in plasma. The fraction <125 μm and the decoction showed better comparative efficacy in bringing ALAT and ASAT activities significantly back to normal. These results show the hepatoprotective effect, which would be due to the presence of bioactive phenolic compounds in the unsieved powders, granulometric fractions, and decoction of *F. dicranostyla* (Yves et al., 2022). These studies corroborate those of Huang et al. (2010); Itoro Usoh et al., (2012) who showed the protective effect of plant extracts on chemical‐induced hepatotoxicity in rats. Bioactive compounds are once again found in the fine fraction (<125 μm).

Generally, polyunsaturated fatty acids, the main components of the membrane lipids, are susceptible to peroxidation. Indeed, CCl_4_ gets metabolized to CCl_3_OO^●^ peroxide free radicals in the liver via mitochondrial cytochrome P_450_ (CYP_450_). These radicals react with proteins or lipids or abstract a hydrogen atom from an unsaturated lipid, thus initiating lipid peroxidation, increasing MDA concentrations (Recknagel et al., 1989). An increase in MDA concentration points out the actions of the toxic metabolite (CCl_4_) which is associated with oxidative stress as a response to free radical production. MDA levels are a pertinent indicator of lipid peroxidation of cell membranes, but also an indicator of the failure of the antioxidant defense mechanism, which is supposed to inhibit high MDA production (Ugwu & Suru , 2021). CCl_4_ significantly increased MDA levels in the negative control group compared with the normal control group. This reflects a state of oxidative stress induced by the reactive metabolites of CCl_4_. Treatment with granulometric classes, unsieved, and decoction of *F. dicranostyla* prevented a significant increase. The fraction with a diameter <125 μm and the decoction again showed better activity. Particle size reduction enhances the accessibility of bioactive compounds, as the process increases the specific surface area of particulate materials, which could improve the antioxidant capacity of plants (Wang et al., 2014). According to Abdus et al. (2016) and Lee et al. (2017), the protection provided by plants against CCl_4_‐induced hepatotoxicity is basically due to the inhibitory nature of the phytochemicals present in them. These phytochemicals can inhibit the microsomal enzymes to restrict the generation of free radicals and stop lipid peroxidation through their antioxidant ability (Molehin et al., 2017). The obtained result is consistent with reports by Mai‐Mbe et al. (2021) who observed a decrease in the MDA concentration following treatment with powder fractions and solvent extracts of *Diospyros mespiliformis* after CCl_4_ injection.

Both SOD and CAT play an important role in defense mechanisms against the harmful effects of reactive oxygen species and free radicals in biological systems. When SOD is an enzyme that converts superoxide radicals (O_2_^●^) to hydrogen peroxide and molecular oxygen, the CAT enzyme decomposes the hydrogen peroxide generated during lipid peroxidation and is also decreased following CCl_4_ induction without treatment with plant extract. The decrease in liver CAT and SOD activities corroborates the above results of MDA concentrations. As a product of lipid peroxidation, the MDA concentration reflects the liver lipid peroxidation level (Ugwu & Suru, 2021). A significant reduction in SOD and CAT activities observed in negative control groups was recorded when animals were treated with the granulometric classes, unsieved, and decoction from *F. dicranostyla* leaf powders. These results could be explained by the fact that the hepatoprotective activity of *F. dicranostyla* leaf powder samples could be due to its free radical scavenging and antioxidant activity, resulting from the presence of certain flavonoids and phenolic compounds in their extracts. Indeed, previous analyses of the particle size of the granulometric classes, unsieved, and decoction from *F. dicranostyla* leaf powders showed the presence of total polyphenols and flavonoids, as well as Zn and Cu as cofactors of the antioxidant enzymes SOD and CAT (Yves et al., 2022). On the other hand, phenolic compounds are more soluble in a liquid matrix than in an aqueous media (Ramadan, 2013). The antioxidant potential of phenolics is mainly due to their redox properties and is the result of various mechanisms: antiradical activity, transition‐metal‐chelating activity, and/or singlet‐oxygen‐quenching capacity (Bettaieb et al., 2010; El‐Hadary & Hassanien, 2016). Other studies on the protective effect of plant powder fractions and solvent extracts against chemical‐induced oxidative stress revealed similar results that are in agreement with the present findings (Soualeh, Stiévenard, Baudelaire, Bouayed, & Soulimani, 2018; Soualeh, Stiévenard, Baudelaire, Soulimani, & Bouayed, 2018).

## 5 CONCLUSION

This study demonstrated the differential effects of powder particle size and decoction powder on the preventive hepatoprotective and antioxidant activities of *F. dicranostyla* against CCl_4_‐induced liver damage. Greater hepatoprotective and antioxidant effects were found with fine powder size as well as decoction powder. Higher liver injury reduction as evident from a decrease in blood plasma lipid profile, namely, CT, TG, LDL‐c, in transaminases (ALAT, ASAT), and lower MDA production was found in treated rats with fine powder fraction of <125 μm, where near and/or similar to those of decoction powder. The highest liver CAT and SOD activities were also observed in rat groups treated with a fine powder fraction of <125 μm, suggesting that treatment with the fine powder, similar to decoction powder has relatively restored the normal levels of lipid status and antioxidant‐related enzymes. Thus, in vivo, studies on animal models prove the bioactivity of *F. dicranostyla* powder fraction from the controlled differential sieving process, thus could be a good alternative to conventional extraction employing solvents.

## AUTHOR CONTRIBUTIONS

**Yves Tabi Omgba:** Conceptualization (lead); formal analysis (equal); methodology (equal); resources (equal); validation (equal); visualization (equal); writing – original draft (equal). **Marthe Valentine Tsague:** Conceptualization (equal); data curation (equal); formal analysis (equal); methodology (equal); resources (equal); validation (equal); writing – original draft (equal); writing – review and editing (equal). **Roméo Joël Temdie Guemmogne:** Conceptualization (equal); formal analysis (equal); methodology (equal); validation (equal); writing – original draft (equal); writing – review and editing (equal). **Achick Estella Tembe:** Conceptualization (equal); project administration (equal); validation (equal); writing – original draft (equal); writing – review and editing (equal). **Rose Ngono Mballa:** Formal analysis (equal); methodology (equal); project administration (equal); writing – review and editing (equal). **Charles N Fokunang:** Conceptualization (equal); project administration (equal); supervision (equal); validation (equal); writing – review and editing (equal). **Bonaventure Ngadjui Tchaleu:** Conceptualization (equal); project administration (equal); supervision (equal); writing – review and editing (equal). **Théophile Dimo:** Conceptualization (equal); project administration (equal); supervision (equal); validation (equal); writing – review and editing (equal). **Judith Ndongo Embola:** Conceptualization (equal); project administration (equal); supervision (equal); validation (equal); writing – review and editing (equal). **Jacqueline Ze Minkande:** Conceptualization (equal); project administration (equal); validation (equal); writing – review and editing (equal).

## FUNDING INFORMATION

This study did not receive any specific grant from any funding agency or scholarship.

## CONFLICT OF INTEREST STATEMENT

All the authors of this manuscript admitted that they have no conflicts of interest with the above findings and research.

## ETHICS STATEMENT

All of the experimental protocols conducted on rats were performed by their animal laboratory use which accepted the principles and was approved by the University Animals Ethical Committee and carried out with approval from the Cameroonian National Ethics Committee (Ref. No FWIRD00001954).

## Data Availability

The data which supported the findings of this study are openly available in the manuscript.
